# Caregiving for a Companion Animal Compared to a Family Member: Burden and Positive Experiences in Caregivers

**DOI:** 10.3389/fvets.2018.00325

**Published:** 2018-12-21

**Authors:** Karysa Britton, Rachel Galioto, Geoffrey Tremont, Kimberly Chapman, Olivia Hogue, Mark D. Carlson, Mary Beth Spitznagel

**Affiliations:** ^1^Department of Psychiatry, Rhode Island Hospital, Providence, RI, United States; ^2^Department of Psychiatry and Human Behavior, Alpert Medical School of Brown University, Providence, RI, United States; ^3^Department of Psychological Sciences, Kent State University, Kent, OH, United States; ^4^Department of Quantitative Health Sciences, Lerner Research Institute, Cleveland, OH, United States; ^5^Stow Kent Animal Hospital, Kent, OH, United States

**Keywords:** caregiver, burden, positive aspects of caregiving, family caregiving, pet caregiving, companion animal

## Abstract

**Introduction:** Research in human caregiving shows burden is often present in the caregiver and can be reduced by interventions that increase positive perceptions of caregiving. Recent work suggests burden is also present in owners of a seriously ill companion animal. To help determine if findings from the human caregiving literature are likely to generalize to companion animal caregiving, we undertook a comparison of burden and positive aspects of caregiving in these groups.

**Material and Methods:** Caregivers recruited through social media disease support and information groups completed self-report questionnaires of burden and positive aspects of caregiving in an online research protocol. Owners of a seriously ill companion animal (*n* = 117) and caregivers of a family member with dementia (*n* = 252) were cross-sectionally compared. Analyses in the full sample were repeated in a subset (*n* = 75 per group) of caregivers with blindly matched demographic profiles.

**Results:** Burden was elevated in both dementia and companion animal caregiver groups, though higher overall for dementia caregivers (*p* < 0.001 for full and matched samples). In contrast, greater positive aspects of caregiving were reported by companion animal caregivers (*p* < 0.001 for full and matched samples). In both groups, positive aspects of caregiving were negatively associated with burden (full sample *p* < 0.001; matched sample *p* < 0.05). Exploratory item analyses suggested the two groups show comparable experiences of fearing the future, guilt, and financial strain (*p* = *ns* for full and matched sample).

**Discussion:** Although both groups showed elevated burden, companion animal caregivers reported less burden and a more positive appraisal of caregiving. Elements of burden showing similarities across groups provide a foundation for understanding caregiver burden in the companion animal owner. The inverse correlation between positive aspects of caregiving and burden suggests the impact of positive caregiving experiences should be considered in burden interventions, but because companion animal owners already positively appraise caregiving, enhancing positive aspects of caregiving may not offset burden as it does in human caregiving samples.

## Introduction

Caregiver burden is a multifaceted reaction of distress to the problems and challenges encountered while providing informal care for someone with an illness (1, 2). This burden encompasses a range of negative experiences present in this context, such as feelings of guilt, anger toward the care recipient, not having enough time to manage responsibilities, fear of what the future holds, financial strain, or feeling that one's health or social life has suffered due to caregiving (1). Burden in caregiving has been linked to adverse emotional states, psychiatric morbidity, and physical, financial, and social repercussions for the caregiver (3). Research demonstrates physiological consequences of burden, including higher daytime cortisol (4, 5), detrimental psychosocial outcomes including anxiety and depression (6, 7), increased risk of mortality for the caregiver (8), and greater likelihood of institutionalization for the care recipient (9). The burden of caregiving has been well-studied in recent decades and is of great public health significance.

Behavioral interventions have been shown to reduce burden and distress in family caregivers (10, 11), with positive aspects of caregiving playing an integral role in outcomes (12). Many different positive aspects of caregiving have been identified in the literature, including emotional satisfaction, such as feeling that providing care makes one feel more useful, needed, appreciated, or confident; personal or spiritual growth; feelings of competency and mastery; relationship gains, role satisfaction, and fulfilling a sense of duty (13–15). A positive appraisal of caregiving is viewed as protective against negative outcomes for both the caregiver and care recipient; when present in the context of caregiving, a positive appraisal of the caregiving experience can give meaning to the caregiver's life and strengthen relationships (16), predicting better health, less depression, and lower burden (12, 13). Importantly, positive aspects of caregiving have been shown to moderate treatment outcomes for burdened caregivers, such that individuals endorsing lower positive aspects of caregiving demonstrate greater benefit from behavioral intervention (12), suggesting that a tendency to positively appraise the caregiving experience may impact the degree to which a caregiver responds to behavioral intervention for caregiver burden.

While the impact of caregiving in human relationships is relatively well-established, this topic has rarely been examined in individuals providing care for a seriously ill companion animal. Over one-third of households in the United States include a dog (36.5%), and nearly as many have a cat (30.4%)^1^. It is common for a companion animal, particularly a dog or cat, to be viewed by the owner as a member of the family (17–19). Although research suggests several health and social benefits of owning a companion animal [reviewed by Cherniack and Cherniack (20)], debate exists regarding the notion that pet ownership is uniformly beneficial (21), and the impact of providing long-term care for a companion animal with medical problems is not well understood. This issue becomes increasingly relevant as advances in veterinary medicine present the option to extend the life of a seriously ill companion animal. Protracted symptom management could be complex and time consuming for the companion animal owner, leading to caregiver burden.

Past qualitative research (22) suggested issues related to caregiver burden were present in a small sample of owners of an aged or chronically ill dog, including greater care needs of the companion animal and related concerns of finances, work, and social life. More recently, measurement of companion animal caregiver burden (23, 24), showed that, compared to those with a healthy companion animal, owners of a dog or cat with a serious illness reported greater caregiver burden and psychosocial distress, including above average levels of stress and clinically meaningful symptoms of depression. Such findings suggest that intervention may be warranted in this population.

The potential for future work translating caregiver burden treatments from human to companion animal caregiver populations will be informed by an appreciation of how caregiving experiences, particularly burden and positive aspects of caregiving, compare. Because prior work in human medicine demonstrates that the degree to which the caregiving experience is positively appraised influences response to intervention for caregiver burden, we sought to understand how burden and positive aspects of caregiving relate and compare in groups of individuals providing care for a relative or a companion animal. We chose dementia caregiving as the comparison sample due to the well-established findings of burden and record of successful interventions in this population. This comparison will provide a foundation for future interventions in companion animal caregivers.

We hypothesized that, consistent with past work, burden and positive aspects of caregiving would be negatively related to each other. To our knowledge, no prior comparisons of this nature have been conducted; as such, we do not have formal *a priori* hypotheses regarding group differences. However, the caregiving experience for these two groups may differ for many reasons, perhaps most notably due to the option of euthanasia for companion animal caregivers. We believe it is plausible that greater burden would be found in dementia caregivers, while positive aspects of caregiving may be greater in companion animal caregivers. Specifically, the companion animal caregiver has made a decision to provide care rather than euthanize for a diagnosis of serious illness. The decision to assume the caregiving role may thus predispose the companion animal caregiver to a more positive caregiving experience. Exploratory item comparisons were also conducted to elucidate any group differences that might inform future research.

## Material and Methods

### Participants

**Figure 1:**
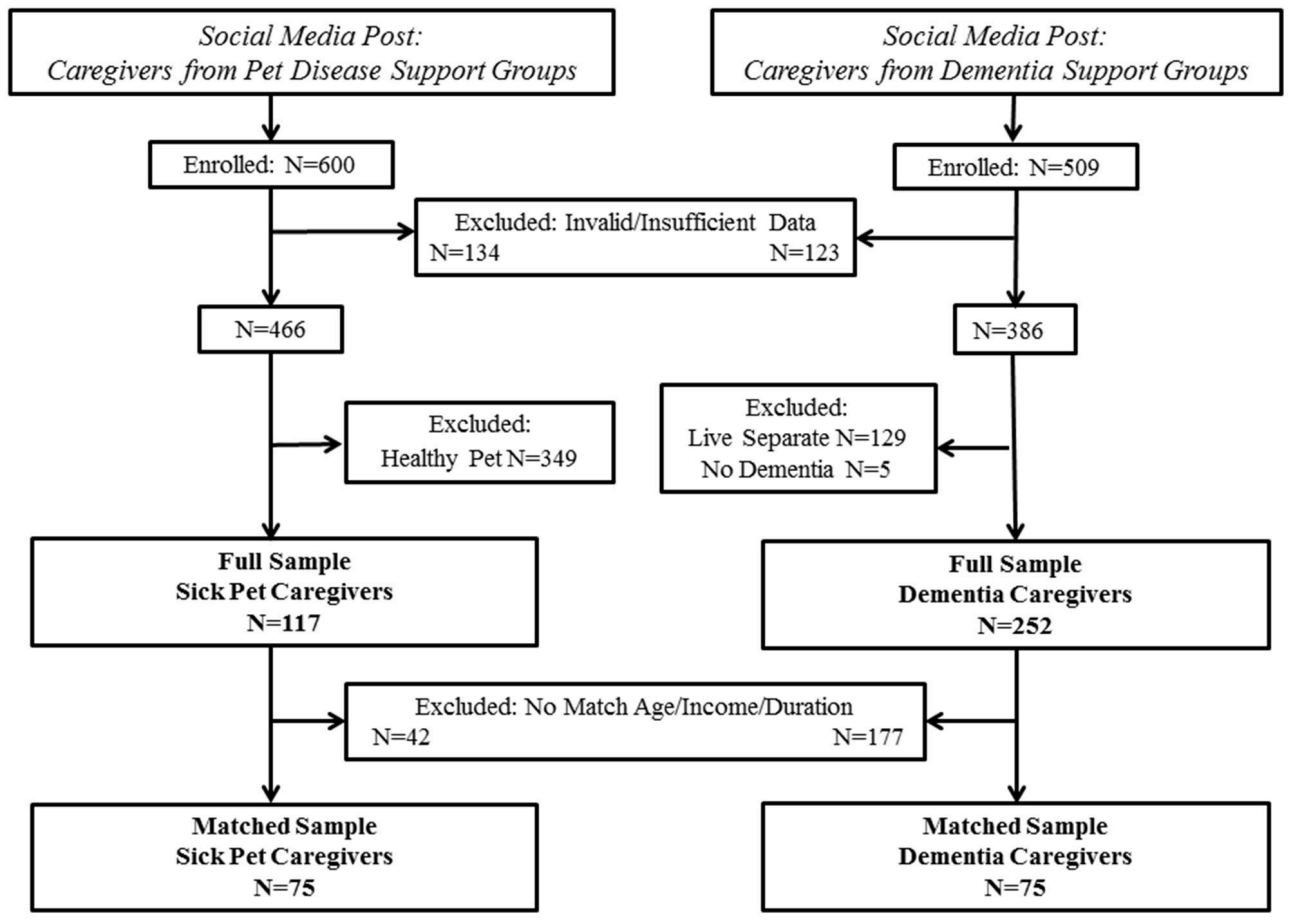
Inclusion and Enrollment.

The present paper draws data from two independent studies with similar methods. Companion animal caregiver data were extracted from an existing dataset that has been previously described (23). New data were collected for the dementia caregiver sample. Inclusion criteria for the current study were that the caregiver must be at least 18 years of age, able to read and comprehend English, and living with/currently providing daily care for the care recipient. Companion animal care recipients were required to be a dog or cat with a current diagnosis of an illness that a veterinarian concurred would be considered a serious illness (i.e., chronic or terminal disease; 23). Dementia care recipients were required to have a diagnosis of dementia given by a physician. Participants not meeting these criteria or having invalid or insufficient data were removed, resulting in the “full sample.” Because demographic differences in age, income, and duration of caregiving were observed in the full sample, a “matched sample” was also created. Specifically, participants were blindly matched without reference to other variables for caregiver age (within 5 years), household income (<$50,000, $50–$100,000, >$100,000), and duration of caregiving (<1 or ≥1 year). Study enrollment/inclusion is shown in Figure 1.

### Measures

#### Demographic Information

Demographic variables of age, gender, education, race, income, and duration of caregiving were assessed via questionnaire.

#### Zarit Burden Interview [ZBI; (1)]

The ZBI assessed caregiver burden. In its original form, the ZBI is a 22-item self-report inventory that asks caregivers to rate the frequency with which they experience the stressful or negative implications of caregiving. This is rated on a 5-point scale ranging from 0 (never) to 4 (nearly always). The ZBI was modified for companion animal caregivers to include 18 items, and the adapted version was recently validated (23). In order to compare groups, just these items were used in the current analyses. Both the total sum and individual item responses were examined in the current study. A summed score above 20 on the original ZBI is considered indicative of clinically meaningful burden (1). Psychometric properties of the original and adapted ZBI include internal consistency of α = 0.82–0.92, test-retest reliability of *r* = 0.88–0.89, and demonstration of convergent validity (1, 23). Cronbach's alpha for the current study was 0.90.

#### Positive Aspects of Caregiving [PAC; (15)]

The PAC is a 9-item scale that assesses positive experiences associated with caregiving on a 5-point scale ranging from 1 (disagree a lot) to 4 (agree a lot), with higher scores representing a more positive appraisal. Total sum and individual item responses were examined in the current study. Psychometric properties include internal consistency of α = 0.89 and construct validity supported through positive correlation with well-being and negative correlation with depressive symptoms (15). Cronbach's alpha for the current study was 0.88.

### Procedures

This research was conducted and reported in accordance with STROBE [Strengthening the Reporting of Observational Studies in Epidemiology; (25)] criteria for cross-sectional studies. Study protocols were approved by the Kent State University Institutional Review Board #16–506 and #17–469. Data were collected during October of 2016 for companion animal caregivers and October of 2017 for dementia caregivers. Recruitment methods for both studies included chain referral and purposive sampling through social media support/information groups (i.e., companion animal disease support/information groups, dementia support/information groups). These methods were employed to help optimize demographic matching for the two samples of interest. Data collection for both protocols was similar. A social media message containing a direct link to the study protocol was posted with open (public) permissions in companion animal disease and dementia support/information groups. The post requested that caregivers anonymously respond to questions about “how taking care of a companion animal affects the owner” (companion animal caregivers) or “how providing care for a loved one with dementia affects the caregiver” (dementia caregivers) and encouraged sharing of the post.

Both online protocols began with informed consent. Respondents were required to acknowledge that participation was voluntary, that responses would be used for research, and that they met inclusion criteria. Consent to participate was given by clicking to advance to the study protocol. Only those providing informed consent were enrolled.

### Power Analysis

Meta-analysis of 84 studies comparing caregiving relatives to non-caregiving relatives of frail older adults showed moderate effect sizes for group differences in caregiver stress (26). Using a significance level of α = 0.05 and power π = 0.8 for a medium effect (δ = 0.5), a minimum sample of 102 individuals was needed (51 per group) according to G^*^Power 3 (27) calculations. As there have been no prior comparisons of these exact issues in companion animal and family caregivers, we intentionally over-recruited for the current study to ensure a sufficient sample with complete data.

### Statistical Analyses

Statistical analyses were identical for full and matched samples unless otherwise indicated. Descriptive statistics and group comparisons were conducted for demographic variables using independent samples *t*-tests (age) and Chi-square analyses (sex, race, education, annual income, caregiving duration, clinically elevated burden [ZBI>20]) to characterize the sample and compare caregiver groups. Variables were evaluated for normality using histograms and skewness/kurtosis values; the PAC and adapted ZBI demonstrated normal distributions in both full and matched samples. Independent samples *t*-tests examined expectations that group differences would emerge in total scores on the adapted ZBI and PAC; degrees of freedom were adjusted for unequal variances when present. To examine the relationship between PAC and ZBI scores within each caregiver group separately, we stratified by group and conducted linear regression analyses, controlling for any demographic variables that displayed significant associations with both PAC and ZBI. In order to determine relevant covariates, bivariate correlations (Pearson for continuous data, Spearman for ordinal data) examining relationships among demographic variables and caregiving measures (i.e., adapted ZBI and PAC) were conducted separately for each group. The familywise alpha level for significance tests was set at 0.05, with application of the sequentially rejective Holm–Bonferroni correction (28) to minimize type I error, with the exception of exploratory item analyses, for which a liberal alpha level was set at 0.05 for each item. All statistical analyses were conducted using SPSS 23.0 (Armonk, NY: IBM Corp).

## Results

### Participant Demographics

In the full sample, significant group differences emerged in age *(t*_(207)_ = −8.54, *p* < 0.001), income distribution [$\chi_{\left(\text{n}=354\right)}^{2}$ = 34.85, *p* < 0.001] and duration of caregiving [$\chi_{\left(\text{n}=354\right)}^{2}$ = 46.04, *p* < 0.001], such that companion animal caregivers reported younger age, greater household income and shorter duration of caregiving. There were no significant group differences for gender, education, or race.

The matched samples showed no differences between groups for age, education, race, gender, income, or length of caregiving. See Table 1 for sample characteristics and group comparisons.

**Table 1:** Sample characteristics and differences between caregiver groups.

	**Full sample**			**Matched sample**		
	**Dementia** **(*n* = 252)**	**Companion** **animals** **(*n* = 117)**	***p***	**Dementia** **(*n* = 75)**	**Companion** **animals** **(*n* = 75)**	***p***
**Caregiver Age (M/SD)**	58.53 (10.30)	47.97 (11.40)	<0.001	51.96 (9.62)	51.44 (10.37)	0.75
**Care Recipient Age (M/SD)**	73.65(10.54)	9.71(4.64)		71.39(10.45)	9.65(4.90)	
**Female (%)**	94.0%	97.4%	0.16	96.0%	97.3%	0.65
**Race (%)**			0.68			0.23
White	93.9%	92.9%		89.3%	94.7%	
Black	2.0%	0.9%		1.3%	0%	
Hispanic	1.2%	1.7%		4.0%	1.3%	
Asian	2.0%	0.9%		4.0%	0%	
Other	2.0%	2.6%		1.3%	4.0%	
**Education (%)**			<0.001			0.98
≤11 years	10.4%	3.5%		5.3%	5.3%	
12 years	32.3%	15.4%		18.7%	20.0%	
13–15 years	21.9%	25.6%		22.7%	28.0%	
16 years	23.0%	30.7%		29.3%	24.0%	
>16	12.4%	24.8%		24.0%	22.7%	
**Household** **Income (%)**			<0.001			1.0
<50K	53.2%	23.9%		34.7%	34.7%	
50–100K	35.0%	43.6%		41.3%	41.3%	
>100K	11.8%	32.5%		24.0%	24.0%	
**Caregiving Duration (%)**			<0.001			1.0
<12months	11.9%	44.0%		32.0%	32.0%	
≥12 months	88.1%	56.0%		68.0%	68.0%	
**ZBI (M/SD)**	39.72(11.57)	25.39(9.59)	<0.001	39.09(1.77)	24.97(9.170)	<0.001
**PAC (M/SD)**	25.92(8.12)	31.44(6.90)	<0.001	25.36(8.49)	32.40 (6.94)	<0.001

*ZBI, adapted Zarit Burden Interview; PAC, Positive Aspects of Caregiving*.

### Comparing Caregiver Type in Burden and Positive Aspects of Caregiving

In the full sample, clinically significant burden was endorsed by 70.9% of companion animal caregivers and 96.8% of dementia caregivers; chi-square analyses demonstrated this difference was significant, [$\chi_{\left(\text{n}=369\right)}^{2}$ = 53.08, *p* < 0.001]. Average burden scores for both dementia (adapted ZBI = 39.72 ± 11.57) and companion animal (adapted ZBI = 25.39 ± 9.59) caregivers were above the cut-off for clinical significance, with dementia caregivers endorsing greater burden *t*_(269)_ = −12.48, *p* < 0.001. On the PAC, companion animal caregivers reported a more positive appraisal of caregiving (31.44 ± 6.90) than dementia caregivers (25.92 ± 8.12), *t*_(263)_, *p* < 0.001.

In the matched sample, 69.3% of companion animal caregivers and 96.0% of dementia caregivers endorsed clinically significant burden, with chi-square analyses demonstrating this difference was significant, [$\chi_{\left(\text{n}=150\right)}^{2}$ = 18.61, *p* < 0.001]. Average scores for both dementia (adapted ZBI = 39.09 ± 11.77) and companion animal (adapted ZBI = 24.97 ± 9.17) caregivers were above the cut-off for clinically significant burden, with dementia caregivers endorsing greater burden on the adapted ZBI *t*_(148)_ = −8.19, *p* < 0.001. Companion animal caregivers reported a more positive appraisal of caregiving (PAC = 32.40 ± 6.90) than dementia caregivers (PAC = 25.36 ± 8.50), *t*_(142)_ = 5.56, *p* < 0.001. See Tables 2, 3 for exploratory adapted ZBI and PAC item comparisons.

**Table 2:** Zarit burden interview items in caregiver groups.

	**Full sample**			**Matched sample**		
**Item**	**Dementia** **(*n* = 252)**	**Companion** **animals** **(*n* = 117)**	***p***	**Dementia** **(*n* = 75)**	**Companion** **animals** **(*n* = 75)**	***p***
Do you feel that because of the time you spend with your (p/r) that you don't have enough time for yourself?	2.95 (0.97)	1.81 (1.09)	<0.001	2.93 (1.08)	1.79 (1.12)	<0.001
Do you feel stressed between caring for your (p/r) and trying to meet other responsibilities for your family or work?	3.00 (0.96)	2.32 (1.10)	<0.001	3.16 (0.97)	2.28 (1.09)	<0.001
Do you feel embarrassed over your (p/r)'s behavior?	1.37 (1.06)	0.68 (1.0)	<0.001	1.31(1.09)	0.64 (0.97)	<0.001
Do you feel angry when you are around your (p/r)?	1.62 (0.89)	0.45 (0.73)	<0.001	1.61(0.91)	0.41(0.68)	<0.001
Do you feel that your (p/r) currently affects our relationships with other family members or friends in a negative way?	1.96 (1.25)	0.97 (1.03)	<0.001	1.92(1.31)	0.85 (1.10)	<0.001
Are you afraid of what the future holds for your (p/r)?	2.97 (1.07)	3.07 (0.98)	0.39	3.01 (1.17)	3.04 (1.01)	0.88
Do you feel strained when you are around your (p/r)?	2.05 (1.01)	1.01 (0.90)	<0.001	1.99(0.94)	1.01(0.92)	<0.001
Do you feel your health has suffered because of your involvement with your (p/r)?	2.38 (1.09)	0.96 (1.01)	<0.001	2.37(1.11)	0.91(0.99)	<0.001
Do you feel that your social life has suffered because you are caring for your (p/r)?	3.00 (1.06)	1.92 (1.29)	<0.001	2.91 (1.16)	1.81 (1.31)	<0.001
Do you feel uncomfortable about having friends over because of your (p/r)?	1.60 (1.40)	0.98 (1.23)	<0.001	1.56 (1.44)	0.89 (1.19)	0.002
Do you feel that you don't have enough money to take care of your (p/r) in addition to the rest of your expenses?	2.30 (1.35)	2.03 (1.28)	0.08	2.09 (1.46)	2.08(1.35)	0.95
Do you feel that you will be unable to take care of your (p/r) much longer?	1.87 (1.16)	0.74 (1.0)	<0.001	1.76 (1.25)	0.77 (0.99)	<0.001
Do you feel you have lost control of your life since your (p/r)'s illness?	2.63 (1.15)	1.39 (1.08)	<0.001	2.55 (1.28)	1.51 (1.07)	<0.001
Do you wish you could leave the care of your (p/r) to someone else?	1.71 (1.24)	0.56 (0.90)	<0.001	1.63 (1.25)	0.56 (0.86)	<0.001
Do you feel uncertain about what to do about your (p/r)?	2.02 (1.08)	1.55 (1.06)	<0.001	1.88 (1.27)	1.44(1.04)	0.022
Do you feel you should be doing more for your (p/r)?	1.91 (1.09)	1.95 (1.14)	0.77	2.01 (1.21)	1.99(1.20)	0.89
Do you feel you could do a better job in caring for your (p/r)?	1.95 (1.13)	1.47 (1.13)	<0.001	2.05(1.16)	1.48 (1.13)	0.003
Overall, how burdened do you feel in caring for your (p/r)?	2.43 (1.17)	1.52 (1.02)	<0.001	2.35(1.24)	1.51 (1.07)	<0.001

*(p/r), companion animal/relative*.

**Table 3:** Positive aspects of caregiving items in caregiver groups.

	**Full sample**			**Matched**		
**Item**	**Dementia** **(*n* = 252)**	**Companion** **animals** **(*n* = 117)**	***p***	**Dementia** **(*n* = 75)**	**Companion** **animals** **(*n* = 75)**	***p***
Providing help to my (p/r) has made me feel more useful	3.14 (1.3)	3.79 (1.08)	<0.001	3.00 (1.46)	3.99 (0.95)	<0.001
Providing help to my (p/r) has made me feel good about myself	2.87 (1.25)	3.82 (0.95)	<0.001	2.69 (1.28)	3.89 (0.92)	<0.001
Providing help to my (p/r) has made me feel needed	3.56 (1.34)	3.87 (1.07)	0.016	3.37 (1.48)	4.01 (1.07)	0.003
Providing help to my (p/r) has made me feel appreciated	2.62 (1.3)	3.53 (1.06)	<0.001	2.53 (1.27)	3.68 (1.09)	<0.001
Providing help to my (p/r) has made me feel important	2.44 (1.21)	3.22 (1.17)	<0.001	2.45 (1.35)	3.29 (1.21)	<0.001
Providing help to my (p/r) has made me feel strong and confident	2.68 (1.25)	3.19 (1.06)	<0.001	2.60 (1.32)	3.36 (1.06)	<0.001
Providing help to my (p/r) has made me appreciate life more	3.64 (1.41)	4.01 (1.0)	0.003	3.75 (1.35)	4.04 (0.85)	0.113
Providing help to my (p/r) has made me feel more positive toward life	2.50 (1.29)	3.16 (1.05)	<0.001	2.51 (1.38)	3.29 (1.01)	<0.001
Providing help to my (p/r) has strengthened my relationships	2.47 (1.34)	2.84 (1.07)	0.005	2.45 (1.46)	2.84 (1.01)	0.061

*(p/r), companion animal/relative*.

### Associations of Burden in Companion Animal and Dementia Caregiver Groups

In the full sample, the adapted ZBI was negatively correlated with the PAC among both companion animal (*r* = −0.25, *p* < 0.01) and dementia (*r* = −0.50, *p* < 0.001) caregivers. There were no significant relationships between primary variables and age, gender, education, race, income status, or length of caregiving.

In the matched sample, the adapted ZBI was negatively associated with the PAC in both companion animal (*r* = −0.24, *p* = 0.04) and dementia (*r* = −0.47, *p* < 0.001) caregiver groups. In the companion animal caregiver sample, burden was negatively associated with income (*r*_s_ = −0.25, *p* = 0.03) while positive aspects of caregiving were associated with duration of caregiving (*r*_s_ = 0.25, *p* = 0.03). There were no such associations among dementia caregivers. See Table 4 for full correlation results. Given that there were no significant associations between any demographic or caregiving variables and both burden and positive aspects of caregiving, no further analyses were needed.

**Table 4:** Correlations among caregiver groups in the full and matched samples.

	**Full sample**										**Matched sample**									
	**Age**		**Education**		**Duration**		**Income**		**PAC**		**Age**		**Education**		**Duration**		**Income**		**PAC**	
**Variable**	**D**	**CA**	**D**	**CA**	**D**	**CA**	**D**	**CA**	**D**	**CA**	**D**	**CA**	**D**	**CA**	**D**	**CA**	**D**	**CA**	**D**	**CA**
Age	–	–	–	–	–	–	–	–	–	–	–	–	–	–	–	–	–	–	–	–
Education	−0.16^*^	−0.23^*^	–	–	–	–	–	–	–	–	−0.22	0.04	–	–	–	–	–	–	–	–
Duration	0.12	−0.03	−0.11	−0.04	–	–	–	–	–	–	−0.25	−0.25^*^	0.08	−0.07	–	–	–	–	–	–
Income	−0.05	−0.19^*^	0.33^†^	0.31^**^	−0.04	0.02	–	–	–	–	0.08	0.06	0.25^*^	0.20	0.13	0.13	–	–	–	–
PAC	−0.04	0.03	−0.02	−0.10	−0.05	0.19^*^	−0.07	−0.10	–	–	0.04	−0.12	−0.02	−0.18	−0.13	0.25^*^	−0.09	−0.06	–	–
ZBI	−0.07	−0.08	−0.01	<0.01	−0.01	−0.01	0.06	−0.12	−0.50^†^	−0.25^**^	0.03	−0.17	−0.17	0.15	−0.02	−0.17	0.05	−0.25^*^	−0.47^†^	−0.24^*^

* p < 0.05,

** p < 0.01,

† *p < 0.001; CA, Companion Animal Caregivers; D, Dementia Caregivers; Duration, Duration of Caregiving; PAC, Positive Aspects of Caregiving; ZBI, Zarit Burden Interview (Revised)*.

## Discussion

The current study compared caregiver burden and positive aspects of caregiving in companion animal and family caregivers. Burden was greater in dementia caregivers but clinically elevated in both groups and significantly related to financial strain in companion animal caregivers. While overall burden was lower for companion animal caregivers, exploration of individual items suggested several similar experiences. Positive aspects of caregiving were negatively correlated with caregiver burden in both groups and were significantly greater in companion animal caregivers. While the two caregiving groups showed demographic differences in the full sample, the matched sample demonstrated that findings were robust.

Differences in burden and positive aspects of caregiving between caregivers of relatives with dementia compared to companion animal caregivers are not surprising for several reasons. Although companion animals are often regarded as part of the family (17–19), they are likely not viewed by most people as fully equivalent to human family members, and there are differences between companion animal and human relationships in attachment (29). Use of dementia caregivers as the human caregiving comparison group may also partially explain differences in caregiver burden, given the possibility of behavioral disturbance and safety risk in this population (6). This notion is supported by the exploratory item analyses showing that dementia caregivers were more likely than companion animal caregivers to experience the feeling that they cannot leave the house. But perhaps the most important difference is the option of euthanasia for the companion animal caregiver. An individual who provides informal care for a family member may do so reluctantly or out of necessity, owing to financial limitations or lack of other supports (30). In contrast, when faced with diagnosis of a serious illness in a companion animal, the owner can decide to euthanize, which may lead to a rarefied group of caregivers predisposed to a more positive experience in caregiving. In other words, degree of choice in assuming the caregiving role is variable in human caregiving relationships, but the companion animal owner has a clear alternative. More research is needed to fully understand the characteristics of companion animal caregivers, but the current work helps lay a foundation for this understanding.

Regardless of differences in overall levels of burden and positive aspects of caregiving, we observed some group similarities. First, caregivers in both groups reported similar financial strain related to caregiving, particularly once income status was controlled in analyses. Finding financial strain in the dementia caregiver group is expected and consistent with past work (31, 32), but also makes sense for the companion animal caregiver. In 2015, the percentage of Americans covered by a single health coverage type was 78.4% (33) in contrast to the <1% of companion animals covered by insurance^2^. With the out-of-pocket expenses of advanced companion animal health care, financial distress in the companion animal caregiver might be anticipated or even greater than in the dementia caregiver. This idea is supported by the link between burden and income in companion animal (but not dementia) caregivers in matched samples. Additionally, caregivers in the two groups reported similar levels of guilt, specifically feeling they should be doing more for their loved one. Such findings have been repeatedly shown in dementia caregivers (34–36), but why do companion animal caregivers feel they are not doing enough? The answer may again relate to difficulty affording treatment or could perhaps be due to time pressures and availability. While laws have been enacted to support family caregivers in many countries, similar protections typically do not exist for the companion animal caregiver. Inability to take time off might lead to providing a lower than desired level of care, in turn contributing to feelings of guilt for the companion animal caregiver. Finally, comparable levels of fear for what the future holds for the loved one were also found in the two groups, further underscoring the presence and importance of emotional burden in companion animal caregiving. Although these item analyses were conducted in an exploratory manner and require replication, the striking similarity in group means for these three ZBI items sits in stark contrast to the highly significant differences observed on other items.

The current work highlights the impact of caregiving for a seriously ill companion animal, with important implications for interventions to decrease caregiver burden in this population. Caregiving literature has documented that both psychotherapeutic and multicomponent interventions tailored to the specific needs of the caregiver may be effective in reducing burden in human caregiving samples (37, 38); research is needed to determine whether such interventions are beneficial for companion animal caregivers as well. Given that positive aspects of caregiving were negatively related to burden, a reasonable path for future work would seemingly be to determine if enhancing this strength reduces burden in companion animal caregivers. However, at least one past work suggests that individuals endorsing few positive aspects of caregiving showed greater benefit from intervention (12). This might mean that the average companion animal caregiver, with an already positive appraisal of caregiving, would not show substantial benefit from standard psychotherapeutic interventions. Specifically, combining findings of the current manuscript with knowledge from prior work, it appears that interventions to enhance positive aspects of caregiving (e.g., finding greater purpose or value in caregiving) may not be needed or particularly useful for pet caregivers in the way that has been suggested for human caregivers (12). Rather, practical interventions to alleviate the daily load of caregiving and increase instrumental support might be more important. This notion aligns with previous suggestions (23) that educational strategies, intentional respite, and skills-based problem-solving should be considered in designing interventions to reduce burden associated with companion animal caregiving.

This work is not without limitations. The companion animal caregiver participants in the current study included individuals providing care for a companion animal with a chronic, but manageable, illness. In contrast, dementia is typically progressive and life-limiting (39, 40). Future research should consider other caregiving groups for comparison. Additionally, although we were able to categorically match groups for length of care (i.e., less than vs. greater than or equal to 1 year), the rapid companion animal lifecycle prevents matching using precise number of years, as this would have forced one of our two groups to be highly artificial. While doing so may have influenced results, as greater length of caregiving is linked to higher burden (41, 42), it would also have reduced the generalizability of our findings. Additionally, the companion animal and dementia caregivers who participated in the current study were recruited through social media, a decision made to enhance group similarity (i.e., utilization of the same recruitment methods). However, the experiences of individuals recruited through social media may not fully reflect the general companion animal and dementia caregiver populations. Recruitment methods could thus have introduced bias into the sample, though recent work (23, 24, 43, 44) suggests patterns of burden are similar across recruitment methods. Other characteristics, including high socio-economic class and relatively homogeneous gender and racial demographics may also influence generalizability of results, and should be considered in future work. Finally, the cross-sectional design of this study is a limit—longitudinal designs that better address causation are an important next step.

Multiple areas of further research may stem from this work. Euthanasia is an available option for the companion animal caregiver, but this does not necessarily mean it is considered an acceptable option to all. An important question to consider in future research of companion animal caregiving may thus be perception of choice in caregiving. It may also be of benefit to investigate specific companion animal caregiving populations that may be especially burdensome, including diagnoses such as canine cognitive dysfunction. Additionally, existing interventions for family caregivers should be examined to determine adaptability for companion animal caregivers. Recent work has begun to pinpoint specific contributors to companion animal caregiver burden (45); continued efforts to delineate the determinants of burden are needed, followed by work to begin establishing appropriate interventions. Finally, it will be important to ascertain if companion animal owners with this presentation should optimally be seen by specialized providers, such as a veterinary social worker, or if this burden may be sufficiently addressed by general mental health providers.

In summary, the current work demonstrates elevated caregiver burden among caregivers both of seriously ill companion animals and of family members with dementia. While overall burden was higher among dementia caregivers, a more positive appraisal of caregiving was seen in companion animal caregivers. Positive aspects of caregiving were negatively linked to burden in both groups. Future research is needed to better understand the characteristics of individuals who choose to provide care for a seriously ill companion animal. The current findings provide a foundation for understanding caregiver burden in the companion animal owner and point to future directions for companion animal caregiver burden intervention research.

## Author Contributions

KB, RG, GT, MC, and MS manuscript conceptualization; MS and KC study methodology and investigation; KB, RG, and KC data curation; RG and OH statistical analysis; and KB, RG, GT, KC, OH, MC, and MS drafting and critical revision of the manuscript.

### Conflict of Interest Statement

The authors declare that the research was conducted in the absence of any commercial or financial relationships that could be construed as a potential conflict of interest.

## Footnotes

^1^U.S. Companion Animal Ownership Statistics. https://www.avma.org/KB/Resources/Statistics/Pages/Market-research-statistics-US-companion animal-ownership.aspx

^2^Walker, M. (2016). Is companion animal insurance worth the cost? http://www.consumerreports.org/companion animal-products/is-companion animal-insurance-worth-cost/
